# Examining Diversity: a Content Analysis of Cancer Depictions on Primetime Scripted Television

**DOI:** 10.1007/s13187-021-02050-x

**Published:** 2021-06-17

**Authors:** Grace Kim

**Affiliations:** Department of Community Health Sciences, Fielding School of Public Health, University of California, Los Angeles (UCLA), 650 Charles Young Drive South, Los Angeles, CA 90095 USA

**Keywords:** Health communication, Entertainment education, Cancer, Health disparities

## Abstract

Entertainment programming provides a unique opportunity for cancer education, particularly for higher risk racial and ethnic minority groups. Cultural relevancy is key to quality narrative cancer communication, and minorities often prefer media content produced by and featuring members of their own cultural in-group. However, little is known about whether cancer depictions or the television programs they appear in are culturally diverse. Using media content analysis, this study aims to assess the cultural diversity of cancer depictions on primetime scripted television to reveal opportunities to improve cancer education through entertainment. Indicators used to assess cultural diversity at the program level and depiction levels were collected. Out of 111 television programs, 10 (9.01%) programs mentioned cancer, from which 37 cancer depictions were identified. However, the majority of cancer depictions involved White patients and White health providers. Depictions of coping and treatment also dominated with less than 10% of depictions discussing cancer prevention. These patterns reveal a missed opportunity in existing cancer narratives on primetime scripted television and a lack of representation of cultural, social, and environmental factors that affect the health of minority communities, who need to hear these messages the most.

## Introduction

Racial and ethnic minorities experience persistent disparities in cancer mortality, survival, and incidence [1]; thus, innovative ways to address these disparities are needed. Researchers have turned to engaging, transporting stories and narratives to help influence individuals’ cancer-related beliefs and behaviors [2, 3]. Entertainment programming, in particular, has the capacity to reach populations experiencing health disparities, such as racial and ethnic minorities, for several reasons [4]. First, some research suggests that racial and ethnic minorities are heavy consumers of television [4, 5]. Second, racial and ethnic minority viewers often regard entertainment television as a source of health information [6] and are also more likely to act on information they learned through television [5, 7]. Thus, identifying how cancer education through entertainment media can be optimized to address cancer disparities becomes salient.

Entertainment education (EE) incorporates health and other educational messages into popular entertainment media with the goal of positively influencing awareness, knowledge, attitudes, and/or behaviors [8]. From its inception, EE has been closely associated with Bandura’s (1986) Social Cognitive Theory (SCT) [9, 10], which describes the effects of role modeling and vicarious learning on self-efficacy and the likelihood of enacting new behaviors [11]. With this focus on role modeling, EE typically uses actors who are ethnically and culturally homogeneous with targeted audiences [12], also referred to as homophily. Although similar external characteristics, such as race, may not be the sole factor contributing to audience identification, SCT suggests audiences that view role models or characters similar to oneself, who model desired behaviors, are more likely to increase self-efficacy and willingness to attempt the behavior than when less similar models are viewed [10]. Thus, characters in EE are developed to be similar to the intended audience to promote greater identification with those characters, with the goal of enhancing narrative persuasion [13, 14].

In this context, cultural relevancy is key to quality narrative cancer communication, and minorities may prefer media content produced by and featuring members of their own cultural in-group [15]. However, research on cancer depictions on television has often focused on the impact of individual cancer storylines focusing on cancer prevention, screening, diagnosis, treatment, coping, and using cancer patient navigators [16–19]. Little is actually known about whether cancer depictions or the television programs they appear in are culturally diverse, which is an important first step to understanding audience identification with cancer narratives, a construct that is central to narrative persuasion theories. Assessing the cultural diversity of cancer depictions found on popular television shows may disclose unintended programming biases and distortions that affect minorities’ access to cancer information in the entertainment media further perpetuating disparities in cancer outcomes. In other words, this study seeks to address whether cancer-related health messages found on popular television are actually reaching the individuals who need to hear these messages the most. Research questions for this study are threefold:

- RQ1. How frequently is cancer portrayed on primetime scripted television?
- RQ2. To what extent are cancer depictions found on primetime scripted television culturally diverse?
- RQ3. What kinds of health messages are promoted in cancer depictions present on primetime scripted television?

## Methods

A media content analysis was conducted to examine cancer depictions on primetime television shows that aired in the 2016–2017 network television schedule. The 2016–2017 network television schedule includes the 5 major US broadcast networks (ABC, CBS, NBC, FOX, and CW) covering primetime hours. The sample was further limited to scripted series, thus excluding variety, reality, newsmagazines, news, and sports, leaving a final sample of 111 programs. To identify television programs with plots involving cancer, the search function was used to review episode summaries on TV Guide (tvguide.com) and Wikipedia (Wikipedia.org) for key words: cancer, tumor, melanoma, lymphoma, leukemia. Programs that mention cancer based on episode summaries were subsequently watched in full, with a particular focus on episodes with cancer mentioned in the online episode summaries.

One coder collected data at both the program and depiction level, requiring two separate, but related codebooks. Codebook development and coding categories were guided by a previous media content analysis of health issues on primetime television [5]. Program level data included title, season, genre, network, number of episodes in the season, and length of show (i.e., half hour, hour). These data were extracted from IMDb (imdb.com), a popular online database for films and television shows that includes information on cast, production crew, plot summaries, ratings, and reviews, and triangulated with the show’s official webpage on the broadcast network. Depiction level data included the type of cancer mentioned, name and demographics (i.e., age group, gender) of the patient, and provider characters, and whether resources were mentioned (i.e., websites, phone numbers). The coder also noted whether the cancer was depicted as a brief mention, dialogue, and minor or major storyline. A brief mention was defined as a passing mention with no further information or comments. Dialogue entailed at least three lines of text, but did not rise to the level of a minor storyline. A minor storyline was identified as a secondary plotline, often carried throughout the entire episode, but not as central to the episode as the major storyline, while a major storyline was defined as a primary focus of the episode [5]. Type of health information conveyed (i.e., prevention, detection, diagnosis, treatment, prognosis, coping) was also coded, allowing for more than one choice since a depiction could involve both diagnosis and treatment.

Indicators used to assess cultural diversity at the program level included demographic characteristics of the main cast and producers, which were defined by those whose names were mentioned in the show credits. Awards or nominations from minority award shows were extracted from the show’s IMDb webpage. To assess cultural diversity at the depiction level, demographics of patient and provider characters involved in the cancer depiction functioned as proxy measures for the more complex concept of homophily: the degree to which audience members perceive themselves as similar to characters in entertainment programs, which has been defined similarly in other studies [20]. Cultural sensitivity issues that appeared in the cancer depiction were also recorded, such as alternative medicine or religion and spirituality. Collectively, program-level data can answer questions regarding the characteristics of the shows that depict cancer, and whether they varied by genre or race/ethnicity of its producers, while depiction level data describe the characters, content, and the types of cancer that are most frequently portrayed.

## Results

### Television Programs

Out of 111 broadcast primetime programs, 10 (9.01%) programs mentioned cancer: *Chicago Justice*, *Chicago Med*, *Doubt*, *Dr. Ken*, *Grey’s Anatomy*, *Jane the Virgin*, *Marvel’s Agents of S.H.I.E.L.D*, *Pure Genius*, *Scream Queens*, and *This is Us* (Table 1). All but one of them were hour-long programs. The majority of programs with cancer depictions were also classified as dramas, but only a third were medical shows. Almost half of the programs were in their first season, with the outlier of *Grey’s Anatomy*, which was in its thirteenth season. Programs ranged from having 1 to 8 cancer depictions, with two medical dramas, *Pure Genius* and *Chicago Med*, exhibiting the most cancer depictions.

**Table 1:** Description of television programs with cancer depictions (N = 10)

Program	Network	Genre	Season	Episodes in season	Number of cancer depictions	Number of minority producers	Number of minority cast	Minority cast/total cast	Number of minority awards or nominations
*Chicago Justice*	NBC	Crime, Drama	1	13	4	0	3	0.60	1
*Chicago Med*	NBC	Drama	2	23	8	0	4	0.44	0
*Doubt*	CBS	Drama	1	13	3	0	3	0.43	0
*Dr. Ken*	ABC	Comedy	2	22	3	1	6	0.75	0
*Grey’s Anatomy*	ABC	Drama, romance	13	24	4	1	6	0.43	2
*Jane the Virgin*	CW	Comedy	3	20	2	0	4	0.57	4
*Marvel's Agents of S.H.I.E.L.D*	ABC	Action, adventure, drama	4	22	1	0	2	0.29	0
*Pure Genius*	CBS	Drama	1	13	8	0	3	0.43	0
*Scream Queens*	FOX	Comedy, horror, mystery	2	13	1	0	3	0.25	0
*This is Us*	NBC	Comedy, drama, romance	1	18	3	0	3	0.38	8

With regard to cultural diversity, 30% of programs with cancer depictions featured minority-dominant casts with more than half of the main cast being of a racial and ethnic minority: *Jane the Virgin*, *Dr. Ken*, and *Chicago Justice*. Of note, *Jane the Virgin* and *Dr. Ken* are shows targeting specific ethnic groups. *Jane the Virgin* is a romantic dramedy on the CW that parodies common tropes of telenovelas, featuring the story of Jane Villanueva, a devout 23-year-old Latina virgin, who becomes pregnant after an accidental artificial insemination. *Dr. Ken* is an ABC sitcom, created, written, and co-produced by its lead actor, Ken Jeong, that follows the story of a Korean American doctor and his family. Two programs involved an executive producer that was of a racial and ethnic minority: Ken Jeong (*Dr. Ken*) and Shonda Rhimes (*Grey’s Anatomy*). Lastly, 40% of programs with cancer depictions were recognized by minority award shows, such as the Black Reel Awards and the National Association for the Advancement of Colored People (NAACP) Image Awards, demonstrating a degree of approval and acknowledgement of how minority groups were represented on the show.

### Cancer Depictions

Across the 10 primetime programs, 181 episodes were reviewed, among which 37 cancer depictions were identified in total (Table 2). Most depictions appeared as minor single-episode storylines, but about a quarter of these depictions only entailed brief mentions of cancer. For example, in a brief mention on *Scream Queens*, the patient (a werewolf) loses all of her hair and compares herself to a cancer patient, prompting the Chanels to give her a makeover. Other examples of cancer depictions from each show are reported in Table 3. The type of cancer featured in these depictions varied widely, with the most common being general cancer of which the type was never identified. The next most frequently mentioned were lung cancer, breast cancer, pancreatic cancer, and leukemia, which were each found three times. Single depictions of cancer included stomach, cervical, kidney, and prostate cancer.

**Table 2:** Description of cancer depictions (N = 37)

Characteristic	N (%)
Role	
Brief mention	9 (24.3)
Dialogue	7 (18.9)
Minor storyline	16 (43.2)
Major storyline	5 (13.5)
Type of health message conveyed	
Prevention	2 (5.4)
Detection	7 (18.9)
Diagnosis	9 (24.3)
Treatment	15 (40.5)
Prognosis	7 (18.9)
Coping	10 (27.0)
Resources	1 (2.7)
Race of cancer patient*	
White	23 (62.2)
Black	9 (24.3)
Latino	1 (2.7)
Asian	3 (8.1)
Biracial	0 (0.0)
Unknown	0 (0.0)
Race of health provider*	
White	14 (37.8)
Black	1 (2.7)
Latino	0 (0.0)
Asian	4 (8.1)
Biracial	1 (2.7)
Unknown	16 (27.8)
Cultural sensitivities	
Religion, spirituality	0 (0.0)
Alternative medicine	1 (2.7)

^*^Percentages may not sum to 100 because not all cancer depictions involved a patient and provider

**Table 3:** Examples of cancer depictions

Program	Cancer depiction
*Chicago Justice*	Officer Ted Cody, a victim of homicide, was struggling financially and battling pancreatic cancer with 6 weeks left. He wanted to ensure that his son would be taken care of after his death
*Chicago Med*	Nurse Maggie’s transgender sister experiences sudden blindness and is diagnosed with prostate cancer
*Doubt*	Sadie discovers that Billy had leukemia and received a blood marrow transplant from cousin Max, meaning that he does not have the same blood as he did when Amy was murdered and that his blood will not match what was found on the murder weapon. This leads Sadie to believe Billy is guilty of the murder
*Dr. Ken*	Allison finds a lump in her breast and receives the results from her mammogram
*Grey’s Anatomy*	Maggie’s adoptive mother is diagnosed with inflammatory breast cancer
*Jane the Virgin*	Rafael explains his motivation for exercising is his history with cancer
*Marvel Agents of S.H.I.E.L.D*	Dr. Radcliffe creates Aida (android version of ex-girlfriend Agnes) and “the Framework” because she was diagnosed with a brain tumor and he promised her a way for her to live
*Pure Genius*	Angie Cheng’s mother diagnosed with cervical cancer and struggles with cultural barriers, but eventually agrees to innovative nanobot treatment
*Scream Queens*	Patient (“werewolf”) loses all of her hair and compares herself to a cancer patient, prompting Chanels to give her a makeover
*This is Us*	William diagnosed with stage 4 stomach cancer and his family learns to cope

Health messages around coping and treatment dominated the cancer depictions found on primetime shows. An example of a treatment storyline on *Chicago Med* featured Haley Cline, a child cancer patient, who experiences complications with infection and liver failure while being treated for non-Hodgkin’s lymphoma. Less than 10% of depictions discussed crucial messages on cancer prevention, such as risk factors or cancer screenings. Only one depiction offered any resources, which was found in an episode of *Dr. Ken* that concluded with a public service announcement (PSA) promoting mammogram screening. With regard to diversity, the majority of cancer depictions involved White patients (62.2%) and White health providers (37.8%), and only one depiction addressed cultural sensitivity issues. *Pure Genius* addressed cultural barriers to healthcare when Angie Cheng’s mother is diagnosed with cervical cancer, but initially refuses to get examined and insists on taking Chinese herbal medicine. When Angie discovers that Dr. Scott Strauss can speak Mandarin fluently, she enlists his help to convince her mother to get examined and eventually accept the innovative nanobot treatment.

## Discussion

The main objective of this study was to assess the cultural diversity of cancer depictions found on primetime scripted television in order to disclose unintended programming biases and distortions that may affect minorities’ access to cancer information in the entertainment media further perpetuating disparities in cancer outcomes. This study found about 10% of broadcast primetime scripted shows in the 2016–2017 network schedule depicted cancer to some extent. This is consistent with previous studies demonstrating the lack of cancer depictions on primetime despite the prevalence of cancer in real life, and how health content tends to lean towards more unusual illnesses and diseases, which make for more dramatic storylines [5]. Findings from the current study thus reaffirm that most health content included in television shows are for entertainment rather than educational purposes [5].

Moreover, study findings reveal a missed opportunity in existing cancer depictions on primetime scripted television to identify with and provide relevant cancer education to minority viewers. Despite some programs having potential to reach minority audiences based on recognition received by minority awards as well as the diversity of its cast and production, the cancer narratives themselves are not actually culturally diverse, mostly consisting of largely White providers and White patients. Based on Social Cognitive Theory, individuals are more likely to pay attention to and be influenced by models who are perceived to be similar [10]; thus, greater degrees of homophily should increase character identification. Through cognitive and emotional processes of character identification, viewers gain a better understanding of the character’s perspective and vicariously participates in the character’s experiences [21]. However, this study finds that the lack of cancer depictions with diverse characters may impede minority viewers from experiencing character identification, thus reducing the impact of narrative persuasion.

Narrative messages also have the potential to achieve effects beyond those directly proposed in EE for role modeling and similarity of external characteristics, such as race. In particular, experiencing identification from the closeness of the story to one’s own situation or culture and subsequent transportation into the story world can reduce counterarguing, or resistance to the message [11, 13, 22]. Thus, the lack of cultural diversity found in cancer depictions also indicates a lack of representation of cultural, social, and environmental factors that affect the health of minority communities. Given that traditional health communication tactics have not adequately addressed diverse populations or health disparities [3], health promotion strategies that address persisting disparities experienced by racial and ethnic minority groups by taking into account complex interrelated psychological, sociocultural, and structural factors are critical [22]. Thus, cancer narratives in entertainment can do much more to not only involve more diverse characters, but better represent the experiences of minority groups for more effective cancer education.

Moreover, storylines on cancer prevention are essential to cancer education efforts, but are largely neglected. Incorporating more cancer prevention narratives is particularly important given racial and ethnic minorities have been documented to be diagnosed later, when the cancer is more advanced [23, 24]. This could be partly explained by low public awareness of cancer prevention strategies, particularly among racial and ethnic minorities [25]. Given the increasing emergence of shows that target minority audiences, such as *Fresh off the Boat*, *Blackish*, *Jane the Virgin*, and *Dr. Ken*, there is a clear opportunity to educate minority viewers about cancer prevention.

### Implications for Practice

Despite the prevalence of cancer in real life, few entertainment programs depict cancer in a meaningful way. A previous study found that cancer is one of the most frequently portrayed illnesses and diseases on medical dramas [6]; however, the current study found that cancer does not have to be limited to medical shows and that cancer narratives can be well integrated into non-medical shows, as exemplified by shows like *This is Us*. Study findings also reveal the discrepancy between current cancer depictions and the groups that need to hear these messages the most. Current cancer depictions on television may unintentionally contribute to persisting cancer disparities that inhibit minority viewers’ access to cancer information on entertainment programming. Not only is there a lack of minority characters in cancer depictions, but when these storylines are presented, they rarely consider the complex, multifaceted cultural factors that are specific to racial and ethnic minority groups. Cancer narratives that can adequately address the social, cultural, environmental, and historical factors affecting a minority group in addition to matching external characteristics of characters may increase receptivity to the storyline [4]. With recent demand for culturally diverse content and movements to diversify Hollywood, there is both monetary and educational opportunity for television networks and producers to include culturally diverse health content that can promote cancer education. Lastly, more spaces for collaboration are needed between public health professionals and entertainment to communicate the needs of minority groups (i.e., prevention) for more effective cancer-related messages on television.

### Limitations

The current study only focuses on broadcast primetime television, but the media landscape is constantly changing, with more entertainment shifting to digital streaming platforms, such as Netflix and Hulu. Future studies should examine how these newer platforms along with social media platforms, such as YouTube, depict and talk about cancer. This study also has some methodological limitations. First, while ensuring inter-rater reliability is ideal, only one coder reviewed the episodes and collected data on cancer depictions. Another limitation is that race and ethnicity of characters is a limited proxy measure of homophily and cultural diversity. External demographic characteristics of characters is only a part of the multidimensional construct of homophily, which also encompasses cultural, social, psychological, environmental, and historical factors that affect the health of a minority community. Lastly, this study was mainly content-oriented with results that have been useful in revealing programming biases and opportunities for cancer education, but sheds little light on how viewers actually make sense of and respond to these narratives. Future research examining the impact of these cancer narratives, particularly the ones that did represent more cultural diversity, may provide additional evidence supporting the need for more culturally inclusive cancer depictions on television.

## Conclusion

Given the tendency to turn to television entertainment for health information, this study reveals a missed opportunity in existing cancer depictions on primetime scripted television. Overall, cancer appears in few programs that feature or are produced by racial and ethnic minority groups. Findings also suggest that the actual cancer depictions often lack cultural diversity with the majority of depictions featuring White characters, indicating a lack of representation of cultural, social, and environmental factors that affect the health of minority communities. Lacking cultural diversity may limit the effectiveness of cancer narratives in reaching minority audiences, who often comprise high-risk groups. Ensuring cancer narratives appear on programs that are appealing to minorities and model culturally sensitive cancer prevention or cancer management behaviors and dialogue may improve reach and audience identification with cancer narratives.

## Data Availability

Not applicable.
